# The Mismeasure of Science: Stephen Jay Gould versus Samuel George Morton on Skulls and Bias

**DOI:** 10.1371/journal.pbio.1001071

**Published:** 2011-06-07

**Authors:** Jason E. Lewis, David DeGusta, Marc R. Meyer, Janet M. Monge, Alan E. Mann, Ralph L. Holloway

**Affiliations:** 1 Department of Anthropology, Stanford University, Stanford, California, United States of America; 2 Paleoanthropology Institute, Oakland, California, United States of America; 3 Department of Anthropology, Chaffey College, Rancho Cucamonga, California, United States of America; 4 Department of Anthropology and Museum of Archaeology and Anthropology, University of Pennsylvania, Philadelphia, Pennsylvania, United States of America; 5 Department of Anthropology, Princeton University, Princeton, New Jersey, United States of America; 6 Department of Anthropology, Columbia University, New York, New York, United States of America

Stephen Jay Gould, the prominent evolutionary biologist and science historian, argued that “unconscious manipulation of data may be a scientific norm” because “scientists are human beings rooted in cultural contexts, not automatons directed toward external truth” [1], a view now popular in social studies of science [2]–[4]. In support of his argument Gould presented the case of Samuel George Morton, a 19th-century physician and physical anthropologist famous for his measurements of human skulls. Morton was considered the objectivist of his era, but Gould reanalyzed Morton's data and in his prize-winning book *The Mismeasure of Man* [5] argued that Morton skewed his data to fit his preconceptions about human variation. Morton is now viewed as a canonical example of scientific misconduct. But did Morton really fudge his data? Are studies of human variation inevitably biased, as per Gould, or are objective accounts attainable, as Morton attempted? We investigated these questions by remeasuring Morton's skulls and reexamining both Morton's and Gould's analyses. Our results resolve this historical controversy, demonstrating that Morton did not manipulate data to support his preconceptions, contra Gould. In fact, the Morton case provides an example of how the scientific method can shield results from cultural biases.

## A Debate across a Century

**Figure 1:**
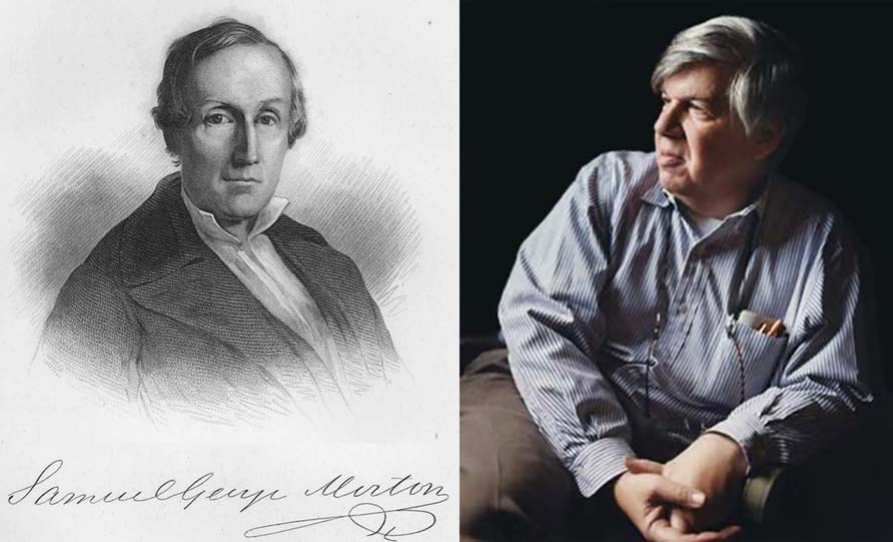
Portraits of Samuel George Morton (left) and Stephen Jay Gould (right). Morton's portrait is from [22] and Gould's photo was taken by Kathy Chapman and is used under a Creative Commons Attribution 3.0 license.

Stephen Jay Gould (1941–2002) and Samuel George Morton (1799–1851) worked in different centuries but shared a number of similarities (Figure 1). Each was well-known to the public and held a prominent academic position, Morton as president of Philadelphia's Academy of Natural Sciences, Gould as a Harvard professor. Gould's popular books on science were best-sellers, and Morton's 1839 *Crania Americana* volume brought him international renown. Both had an exceptionally broad range of research interests that included invertebrate paleontology—Morton was the first American practitioner of this discipline [6], and it was with studies of fossil land snails that Gould initially made his mark [7]. But it was Morton's work on human skulls that drew first Gould's interest, then his ire.

**Figure 2:**
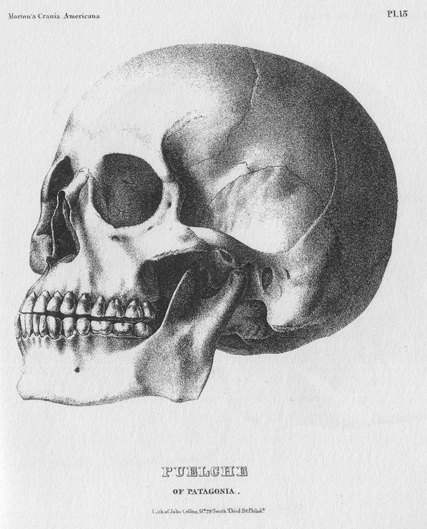
Skull illustrated in Samuel George Morton's *Crania Americana* [**8**]. The 78 lithographs in *Crania Americana* set new standards for accuracy in anatomical illustration, as they were drawn carefully to scale and rechecked for accuracy multiple times, chiefly by John Collins [12],[13]. Indeed, the quality of the illustrations in this volume exceeds that of many modern publications. The remaining specimens in the Morton Collection are currently curated at the University of Pennsylvania Museum of Archaeology and Anthropology.

Much of Morton's fame derived from his “American Golgotha”—a collection of nearly 1,000 human skulls (Figure 2) he obtained from around the world [6]. Morton took detailed measurements of these skulls with a particular focus on cranial capacity, the skeletal equivalent of brain size [8]–[10]. From these measurements he hoped to determine whether different human populations were separate species resulting from multiple divine creations (polygenesis) or a single species created but once (monogenesis), a major question in pre-Darwinian science [6]. Morton's empirical approach, generating data by systematically measuring large numbers of actual specimens, was groundbreaking and he was considered the objectivist of his era [1],[6]. Even so, as the polygenesis-monogenesis debate faded, Morton's work lapsed into relative obscurity [6],[11].

Morton again drew wide attention when Stephen Jay Gould used his skull research as a case study, first in a 1978 *Science* paper [1] and then in his 1981 book *The Mismeasure of Man* [5]. But this was no benevolent rescue from the backfiles of history. Gould reexamined Morton's data on cranial capacity variation in modern human populations and concluded that Morton had selectively reported data (see Box 1), manipulated sample compositions (see Box 2), made analytical errors, and mismeasured skulls in order to support his a priori views on intelligence differences between human groups. When properly analyzed, Gould argued, Morton's measurements show only trivial differences between populations. Gould used Morton as a case study to argue that “unconscious or dimly perceived finagling, doctoring, and massaging are rampant, endemic, and unavoidable in a profession that awards status and power for clean and unambiguous discovery” [1]. Gould's analysis of Morton is widely read, frequently cited, and still commonly assigned in university courses [11]–[13]. Morton has become a canonical example of scientific misconduct and an oft-told cautionary tale of how human variation is inevitably mismeasured.

**Box 1. Did Morton selectively report his data?** Morton divided his skull collection into broad racial groups, such as Native Americans, Caucasians, and so forth, but then also identified specific populations within those broad groups. So Morton's “Indian” (Native American) sample was composed of approximately 28 subsamples from more specific populations, such as Peruvians, Iroquois, “Eskimaux,” and so forth [8]. One of Gould's best-known charges against Morton is that Morton was biased in his reporting of the cranial capacity averages for these subsamples: “It is intriguing that Morton often reported Caucasian means by subsamples, which permitted him to assert the superiority of Teutons and Anglo-Saxons. But he never broke down the Indian mean.…Thus, the fact that some Indian subsamples (Iroquois at 91.5 in^3^, N = 4) exceeded the mean for Americans of Anglo-Saxon stock remained hidden in his raw data. (Morton never calculated the Indian subsample means at all; I have recovered them from his data.)” [1].

But Gould's claim, which has been repeated numerous times, is false. Morton routinely reported “Indian” subsample means, doing so at least 12 times in *Crania Americana*, the publication referenced by Gould. The subsample means reported by Morton include that of the Iroquois, which Morton noted was “within two inches of the Caucasian mean,” as well as that of the “Eskimaux,” which Morton noted was “a near approach to the Caucasian mean” [8]. In fact, Morton reported Native American population means more often than he did for other groups, and the means he reported are representative of his Native American sample as a whole.

**Box 2. Did Morton manipulate his samples?** Gould states that “as a favorite tool for adjustment, Morton chose to include or delete large subsamples in order to match grand means with a priori expectations” [1]. This criticism stems from the fact that each of Morton's broader racial samples (e.g., “Indian”) were composed of multiple population subsamples, typically with differing mean cranial capacities. Thus it is possible to alter the overall “race” means by manipulating their constituent subsamples, and Gould charges that Morton did just that in order to obtain the results he expected.

For example, Gould compares the cranial capacities in Morton's 1839 and 1849 publications and finds that “Morton's Indian mean had plummeted to 79 in^3^.… But, again, this low value only records an increasing inequality of sub-sample size. Small-headed (and small-statured) Peruvians had formed 23 percent of the 1839 sample; they now made up nearly half the total sample” [1]. However, the “Indian” mean was 79.6 in^3^ in Morton 1839 and 79.3 in^3^ in Morton 1849, so the “plummet” Gould refers to was all of 0.3 in^3^. More importantly, Morton in 1849 [10] explicitly calculated his overall “Indian” average by taking the mean of three subgroups: Peruvians, Mexicans, and “Barbarous Tribes”—this is readily apparent in Morton's table reprinted in Gould [1]. As such, the percentage of the overall “Indian” sample composed of Peruvians is irrelevant to the overall mean, as it is only the Peruvian average which impacts the overall value. The Peruvian average changed by less than 1 in^3^ from Morton 1839 (*n* = 33) to Morton 1849 (*n* = 155).

Clearly, Morton was not manipulating samples to depress the “Indian” mean, and the change was trivial in any case (0.3 in^3^). In fact, the more likely candidate for manipulating sample composition is Gould himself in this instance. In recalculating Morton's Native American mean, Gould [1] reports erroneously high values for the Seminole-Muskogee and Iroquois due to mistakes in defining those samples and omits the Eastern Lenapé group entirely, all of which serve to increase the Native American mean and reduce the differences between groups.

## The Current Study

The importance of the Morton case led us to reexamine the fundamental underlying question: did Morton allow his a priori views on human variation to impact the data and analyses he published, as Gould argues? This hypothesis had remained essentially untested for 30 years. While some had raised questions about Gould's characterization of Morton [11]–[13], only one short publication by Michael [14] considered the primary data (Text S1). Unfortunately, the Michael study has multiple significant flaws rendering it uninformative (Text S1). It is rarely cited and, as noted by Kitcher, “virtually nobody has reacted to Michael's article by seeing it as a refutation of Gould” [15].

To test Gould's claim that Morton fudged his data, we relocated and remeasured almost half of the skulls that Morton had originally measured (Text S2, Dataset S1, Dataset S2). Gould did not measure nor personally examine the skulls in the Morton Collection—his argument was based on analyzing Morton's measurements. We also reanalyzed Morton's data and reexamined Gould's evaluation, drawing in part on the Stephen Jay Gould Archive recently made available. Our full analysis, along with all raw data, is given in the Supporting Information section (Text S1, Text S2, Dataset S1, Dataset S2, Dataset S3).

In reevaluating Morton and Gould, we do not dispute that racist views were unfortunately common in 19^th^-century science [6] or that bias has inappropriately influenced research in some cases [16]. Furthermore, studies have demonstrated that modern human variation is generally continuous, rather than discrete or “racial,” and that most variation in modern humans is within, rather than between, populations [11],[17]. In particular, cranial capacity variation in human populations appears to be largely a function of climate, so, for example, the full range of average capacities is seen in Native American groups, as they historically occupied the full range of latitudes [18]. It is thus with substantial reluctance that we use various racial labels, but it is impossible to discuss Morton and Gould's work without using the terms they employed.

## Remeasuring Morton's Skulls

Morton initially measured cranial capacity by filling skulls with seed, but he grew dissatisfied with the accuracy of this method and switched to using lead shot, which yielded more repeatable capacity values [8],[10]. In Morton's initial seed-based 1839 study, “Caucasians” had the largest average cranial capacity (87 in^3^) followed by “Mongolians [Asians]” (83 in^3^), “Malays [Island Southeast Asia]” (81 in^3^), “[Native] Americans” (80 in^3^), and “Ethiopians [Africans]” (78 in^3^) [8]. His final shot-based tally in 1849 again had “Caucasians” with the largest mean capacity (92 in^3^) followed by “Malays” (85 in^3^), the “Negro Group” (83 in^3^), and the “[Native] American Group” (79 in^3^) [10].

Gould famously suggested that Morton's measurements may have been subject to bias: “Plausible scenarios are easy to construct. Morton, measuring by seed, picks up a threateningly large black skull, fills it lightly and gives it a few desultory shakes. Next, he takes a distressingly small Caucasian skull, shakes hard, and pushes mightily at the foramen magnum with his thumb. It is easily done, without conscious motivation; expectation is a powerful guide to action” [5]. While Gould offers this as only a “plausible scenario,” and did not remeasure any crania, subsequent authors have generally (and incorrectly) cited Gould as demonstrating that Morton physically mismeasured crania (e.g., [15]).

We remeasured 308 of the 670 skulls (46%) whose capacity was published by Morton (Text S2, Dataset S1, Dataset S2). Linear and quantile-quantile regression identified Morton's measurements of 7 skulls (2%) as differing significantly from ours (Table 1), with a percentage difference in measurements of greater than 5.5% (Text S2). If Gould's hypothesis that Morton physically mismeasured some skulls due to racial bias were correct, we would expect the mismeasured crania to be non-randomly distributed by population. Specifically, we would expect Morton's overestimates to be concentrated on “white” crania, whereas his underestimates would be mostly “non-white” crania. We tested this using the binomial probability on population-quantile tables (Text S2) and found only one significant difference: Morton overestimated more Egyptian crania (3 of 13) than would be expected by chance. The overmeasured Egyptian skulls are specimens that Morton considered clearly “Negro,” so his overestimation is obviously at odds with his predicted bias. Otherwise, Morton's errors were random with respect to population. Individually, Morton's three most overmeasured skulls are an Egyptian Copt that Morton considered “Negro” (+12%), a Seminole (+8%), and a “Native African Negro” (+7%). These results falsify the claim that Morton physically mismeasured crania based on his a priori biases.

**Table 1:** Crania mismeasured by Morton with shot [10], using our measurements as the “gold standard.”

Specimen #	Population	Cranial Capacity (in^3^)		Difference	Measure Error
		Current	Morton		
761	Egyptian Copt	76	85	+12%	0.5%
754	Seminole	82	89	+9%	0.2%
994	Native African	71	76	+7%	0.4%
1435	Aymara	70	66	−6%	0.3%
949	Arickaree	80	75	−6%	0.2%
1326	Aymara	83	75	−10%	0.5%
70	Chetimaches	84	75	−11%	0.5%

Our capacity measurements (“Current”) have been adjusted to account for the average difference (about 4%) produced by the difference in our method versus Morton's shot method (see Text S2). “Difference” is Morton's measurement relative to ours. Specimens with a percentage difference of greater than 5.5% (more than 2.5 standard deviations from the mean percentage difference) are clear outliers and we consider them to have been mismeasured by Morton. “Measure Error” is our measurement error based on three repeated measurements of each cranium's capacity.

## Seeds, Shot, and Bias

Gould's claim that Morton had mismeasured crania based on race derived from his comparison of Morton's seed-based and lead shot–based measurements, with different races experiencing different changes in their average cranial capacity between the two methods [1]. Gould reconstructs that in going from Morton's seed measurements to shot measurements the average capacity for different groups experienced different increases: 5.4 in^3^ for Morton's black sample, 2.2 in^3^ for his “Indian” sample, and just 1.8 in^3^ for his Caucasian sample. Gould concludes that “surely something funny is going on here.…I strongly suspect a systematic bias for undermeasurement of black skulls [during the initial seed-based measurements]” [1]. This is the evidence Gould offers in support of his “plausible scenario” that Morton may have physically mismeasured crania.

Morton only reported individual seed-based measurements for “Indian” crania, as they were the focus of his 1839 *Crania Americana* volume. Gould derived the “seed to shot” changes in Morton's other samples by making guesses about which skulls had been included, rendering those values highly questionable (Dataset S3). For “Indian” specimens, however, the seed and shot measurements of specific crania can be compared directly. Gould made those comparisons and reports that the average increase from seed to shot is 2.2 in^3^ [1]. But the average, the only result reported by Gould, is deceptive. We found that the changes from seed to shot measurements of individual crania range from an increase of 12 in^3^ to a decrease of 10 in^3^, with a standard deviation of 2.8 (Dataset S3). These increases and decreases do not appear to be patterned by group. For example, one Peruvian cranium increases in capacity by 12 in^3^ (+18%), while another Peruvian cranium decreases in capacity by 5.5 in^3^ (−7%). This casts significant doubt on the hypothesis that mismeasurements with seed were a function of Morton's racial bias.

Rather than bias, the source of changes between Morton's seed-based and shot-based cranial capacities is more likely that stated by Morton himself: mistakes in the seed measurements. The seed-based measurements reported in *Crania Americana* were done in part by an assistant whom Morton later found had made errors. Morton, in describing his 1849 shot-based measurements, stated, “All the measurements in this Catalogue [1849], both of the facial angle and internal capacity, have been made with my own hands. I at one time employed a person to aid me in these elaborate and fatiguing details; but having detected some errors in his measurements, I have been at the pains to revise all that part of the series that had not been previously measured by myself. I can now, therefore, vouch for the accuracy of these multitudinous data…” [10].

## Reevaluating Gould's Analysis

Gould also performed his own analysis of Morton's cranial capacity data and came to the conclusion that “there are *no* differences to speak of among Morton's races” ([1], italics in original). For Morton's 1839 seed-based measurements, Gould claims that Morton's Native American average capacity is artificially depressed by his inappropriate use of a straight mean (taking the average of each individual specimen in the entire sample) rather than a grouped mean (first taking the average of each Native American population subsample, then calculating the mean of those means), since the former is sensitive to differences in sample sizes between “large headed” populations and “small headed” populations. In fact, the grouped mean for Morton's Native American dataset is 79.9 in^3^, almost identical to the straight mean of 80.2 in^3^ (Dataset S3). So Morton's use of a straight mean actually slightly increased his Native American average. Gould's calculation of a higher Native American average (83.8 in^3^) is entirely a function of Gould omitting 34 crania (of 144) as coming from populations with samples of *n*<4 and, even by that criterion, erroneously excluding 6 crania, all with small cranial capacities (Dataset S3).

Gould's reanalysis of Morton's 1849 shot-based data resulted in a Native American mean capacity of 86 in^3^ rather than Morton's original 79 in^3^ [1]. Gould obtained his new average by again taking the group mean of Native American populations with four or more crania. But Gould also applied an additional restriction: he only included Native American crania that Morton had also previously measured with seed. This restriction is entirely arbitrary on Gould's part, as Morton's publications and analyses for his seed- and shot-based measurements are completely separate (1839 versus 1849), and Gould did not apply this restriction to the other groups he reanalyzed in Morton's shot-based data. If this restriction is lifted, Gould's Native American average would be reduced to about 83 in^3^, considerably below his reported 86 in^3^ (Dataset S3).

Overall, Gould concludes that his reanalysis of Morton's shot-based data produces the “remarkable” result that there are no notable differences in mean cranial capacity between Morton's groups, with Caucasians firmly mid-pack at 85 in^3^ and the overall range being 83 to 86 in^3^ [1]. However, Gould's Caucasian figure was in error and should really be 87 in^3^ rather than 85 in^3^ [5]. And even accepting Gould's inflated mean for Native Americans of 86 in^3^, the overall rank order of Gould's results (whites/Native Americans/“Mongolians” and “Malays”/blacks) is then actually closer to Morton's presumed a priori bias than were Morton's own results (whites/“Malays”/blacks/“Mongolians”/Native Americans).

## Our Verdict

Our analysis of Gould's claims reveals that most of Gould's criticisms are poorly supported or falsified. It is doubtful that Morton equated cranial capacity and intelligence [6],[13], calling into question his motivation for manipulating capacity averages. Morton did not consider the influence of sex or stature on cranial capacity, but it would have been impossible for him to use those parameters to bias the averages he reported (see Box 3). The grouped mean of Morton's Native American sample is almost identical to the straight mean, rendering irrelevant Morton's decision to use the latter. The changes in average cranial capacity from Morton's seed-based measurements to shot-based measurements cannot be reconstructed with any certainty, incorporate erroneous seed measurements made by Morton's assistant, yielded a broad range of changes (−10 to +12 in^3^) hidden by Gould's mean, and are confounded by the shifts in sample composition (circa 50%) between the two rounds of measurement. Morton did not manipulate his samples to influence the average cranial capacities, at least not in a detectable manner. Morton did report subsample means for non-Caucasian groups (see Box 1). Of the approximately seven minor errors in Morton's work identified by Gould [1], only two appear to be actual errors, and their overall impact confounds rather than supports Morton's presumed a priori rankings.

**Box 3. Did Morton use sex to skew his results?** Gould faulted Morton for failing to divide his samples by sex when calculating cranial capacity averages, given that differences in mean stature between males and females typically produce attendant differences in mean cranial sizes [1]. Certainly, more accurate population averages would be obtained if each sample were composed of equal numbers of males and females. But the question at hand is whether Morton manipulated his data to fit his preconceptions. In this regard, it is essentially impossible for Morton to have exploited sexual differences in cranial capacity to alter population averages. The only way Morton could have done so is by including more females for the populations he considered “inferior” and more males for “superior” populations. However, Morton did not collect the skulls himself [1],[6], and there is no evidence that he excluded any skulls from measurement based on sex. Indeed, Morton was largely blind to the sex of the skulls in his collection because of the low accuracy of determining sex from the skull, a low accuracy noted as well by Gould [1]. Furthermore, given that Morton's procedure for estimating sex from skulls almost certainly depended heavily on size (as noted by Gould, and as even modern methods do), it is entirely unsurprising to find a notable difference in size between “males” and “females.” Gould faults Morton for failing to notice this difference that “stared him in the face,” but had Morton commented on it he could rightly have been criticized for circularity—assigning sex based on size guarantees that “males” will appear larger than “females.”

Of the substantive criticisms Gould [1] made of Morton's work, only two are supported here. First, Morton indeed believed in the concept of race and assigned a plethora of different attributes to various groups, often in highly racist fashion. This, however, is readily apparent to anyone reading the opening pages of Morton's *Crania Americana*. Second, the summary table of Morton's final 1849 catalog [10] has multiple errors (Dataset S3). However, had Morton not made those errors his results would have more closely matched his presumed a priori bias (and see Box 4). Ironically, Gould's own analysis of Morton is likely the stronger example of a bias influencing results [11].

**Box 4. Did Morton ignore his mistakes?** Gould [1] found that in the final table of Morton's main work, *Crania Americana*, Morton had erroneously reported the Native American mean cranial capacity as 82.4 in^3^ rather than the true value of 80.2 in^3^. As Gould describes, “this elementary error permitted Morton to retain the conventional scale of being with whites on top, Indians in the middle, and blacks on the bottom” [1]. Gould argued that the error persisted because its “demotion” of blacks “provided so much satisfaction that Morton never thought of checking himself” [1]. However, the correct value is given on the page in *Crania Americana* preceding the table in question, suggesting the error in the table was typographical. Furthermore, historical evidence indicates that Morton did check himself and attempt to correct the error.

Michael [14] describes a copy of *Crania Americana* inscribed by Morton with the erroneous “82” value for “Indians” corrected in the same pen to read “80.” A different Morton-inscribed copy of *Crania Americana* reprinted by Bernasconi [21] has the same correction. We found that Gould's personal copy of a first edition *Crania Americana*, while lacking an inscription from Morton, also has the identical correction in ink clearly of considerable antiquity (Gould Archive, Stanford University). In addition, Stanton [6] reproduces the same table with the correct value of 80 set in type. This suggests that a systematic effort to correct this error was made around the time of publication, casting doubt on Gould's claim that Morton “never thought of checking himself.”

Finally, this error did not “demote” blacks: the rank ordering of groups by average cranial capacity remains “White/Indian/Black” whether “Indians” are 80 in^3^ or 82 in^3^. As such, the error does not alter the “scale of being” whatsoever, contra Gould, falsifying the alleged motivation for Morton's error.

It should be noted that, were Gould still alive, we expect he would have mounted a defense of his analysis of Morton. We are saddened that his passing precludes such an exchange. While we differ with Gould in regards to his analysis of Morton, we find other things to admire in Gould's body of work [19]–[20], particularly his staunch opposition to racism [5]. We trust that Gould, having reevaluated the work of Morton long after Morton's passing, would find our reevaluation of “Gould on Morton” an appropriate exercise, even if he would likely have differed with our conclusions.

## Biased Scientists Are Inevitable, Biased Results Are Not

Samuel George Morton, in the hands of Stephen Jay Gould, has served for 30 years as a textbook example of scientific misconduct [12]. The Morton case was used by Gould as the main support for his contention that “unconscious or dimly perceived finagling is probably endemic in science, since scientists are human beings rooted in cultural contexts, not automatons directed toward external truth” [1]. This view has since achieved substantial popularity in “science studies” [2]–[4]. But our results falsify Gould's hypothesis that Morton manipulated his data to conform with his a priori views. The data on cranial capacity gathered by Morton are generally reliable, and he reported them fully. Overall, we find that Morton's initial reputation as the objectivist of his era was well-deserved.

That Morton's data are reliable despite his clear bias weakens the argument of Gould and others that biased results are endemic in science. Gould was certainly correct to note that scientists are human beings and, as such, are inevitably biased, a point frequently made in “science studies.” But the power of the scientific approach is that a properly designed and executed methodology can largely shield the outcome from the influence of the investigator's bias. Science does not rely on investigators being unbiased “automatons.” Instead, it relies on methods that limit the ability of the investigator's admittedly inevitable biases to skew the results. Morton's methods were sound, and our analysis shows that they prevented Morton's biases from significantly impacting his results. The Morton case, rather than illustrating the ubiquity of bias, instead shows the ability of science to escape the bounds and blinders of cultural contexts.

## Supporting Information

Text S1 Additional historical background.
(DOC)

Text S2 Materials and methods.
(PDF)

Dataset S1 Morton's raw cranial capacity data from his three major publications, *Crania Americana* [8], *Crania Aegyptiaca* [9], and *Catalogue of Skulls* [10], in Microsoft Excel format.
(XLS)

Dataset S2 The raw cranial capacity data of the present study, in Microsoft Excel format.
(XLS)

Dataset S3 The analytical spreadsheets showing the calculations described, in Microsoft Excel format.
(XLS)

## Abbreviations

in^3^: cubic inches
