# Correction: The Mismeasure of Science: Stephen Jay Gould versus Samuel George Morton on Skulls and Bias

**DOI:** 10.1371/annotation/138c7c99-249f-432c-a4f7-2993b7b87c0a

**Published:** 2011-07-07

**Authors:** Jason E. Lewis, David DeGusta, Marc R. Meyer, Janet M. Monge, Alan E. Mann, Ralph L. Holloway

The Academic Editor associated with this article was inadvertently omitted. The Academic Editor is: David Penny, Massey University, New Zealand. 

