# Supplementary material for: The Mismeasure of Science: Stephen Jay Gould versus Samuel George Morton on Skulls and Bias
Source: PLoS Biol. 2011 Jun 7;9(6):e1001071. doi: 10.1371/journal.pbio.1001071 (PMC3110184; doi:10.1371/journal.pbio.1001071)
Supplement: Text S1 — Additional historical background. (DOC) [file pbio.1001071.s001.doc]

**TEXT S1: ADDITIONAL HISTORICAL BACKGROUND**

Philadelphia physician Samuel George Morton (1799-1851) was one of the most famous scientists of the 19th century [1], and is often considered both the first American physical anthropologist [2-4] and invertebrate paleontologist [1]. An obituary noted that, “probably no scientific man in America enjoyed a higher reputation among scholars throughout the world, than Dr. Morton” [1], and in life his work attracted praise from prominent scientists [1]. Morton began accumulating human crania in 1830 to use in his lectures on anatomy [5]. The crania were obtained from all corners of the globe via Morton’s friends and admirers [1]. Morton himself did no fieldwork or collecting of his own [1,6].

Morton’s first major work on his cranial collection was *Crania Americana* [7] in which he considered Native American origins and relationships. This was then a high-profile question given the debate over the unity of humankind: multiple species and creations, polygenesis, versus a single species and creation, monogenesis [1,8]. Based on a dozen measurements, most especially cranial capacity, of 158 Native American crania, he concluded that, “the American nations, excepting the Polar tribes, are of one Race and one species, but of two great families ....” [7]. Environmental causes of variation in humans were discounted, and the antiquity of races confirmed [7]. By his own later admission[[1]](#footnote-2), Morton here gave only cautious and indirect support to polygenesis [1,3].

*Crania Americana* received immediate and positive attention [2,4], and was accepted in scientific circles as evidence for the general unity of Native Americans as well as for polygenesis[1]. Morton was particularly acclaimed for his marshalling of actual data (cranial metrics), rather than assertions supported by only anecdotal evidence, as was then more common [1,6]. While an appendix on phrenology by George Combe is included in *Crania Americana*, Morton remained cautious as to the validity of this approach and never used such methods himself [1,2-4].

Morton’s next major publication on skulls was *Crania Aegyptiaca* [9], based on his analysis and measurements of 100 crania from ancient Egypt. Morton concluded that the ancient Egyptian “race” was a mix of different groups, including the “Negro,” but also stated that “unmixed Negroes” in ancient Egypt were servants and slaves [9]. *Crania Aegyptiaca* found quick acceptance among scientists and substantial popularity [1]. For anthropologists of the day, *Crania Aegyptiaca* demonstrated the great antiquity of human races [1]. While Morton himself, “nowhere betrayed interest in the political implications of his researches,” [1], *Crania Aegyptiaca* was immediately hailed in the American South as support for slavery[[2]](#footnote-3) [1].

Morton’s last publication on crania was his 1849 *Catalogue of Skulls of Man* [5] which compiled the capacities of 670 crania. As the title indicates, this is primarily a catalog with little interpretation, as Morton was, “engaged in a memoir which will embrace the detailed conclusions that result from these data,” [5]. Morton died before completing the interpretive work, though portions were published by his colleague Meigs [10]. The collection Morton assembled in his lifetime included 967 human crania, of which 670 are associated with published cranial capacities measured by Morton. The Morton Collection of Crania of Man and the Inferior Animals currently consists of approximately 1400 crania curated at the University of Pennsylvania Museum of Archaeology and Anthropology [11]. The collection currently includes crania from circa 73 global populations, typically with relatively small samples from each population (mean is 9 individuals, range is 1 to 155).

In his time, Morton was noted for providing cranial data in support of polygenesis, the antiquity of races, the unity of (non-artic) Native Americans, the identification of the Moundbuilders as Native Americans, and the identification of the ancient Egyptians as being (mostly) Caucasian with “Negro” slaves [1,2-4]. These findings were taken up by the polygenesis movement, and the *Types of Mankind* volume [12] credited him as the prime inspiration. Morton was also considered the objectivist of his age based on the data he had amassed and the primacy he assigned to it [6]. Morton’s work influenced Broca [2,13], who in turn influenced Hrdlicka [14], who noted the importance of Morton, tabbing him as the first American physical anthropologist [4]. Morton’s work initiated the use of anatomical measurements to compare populations [2], and also pioneered the scientific illustration of skulls [3,4].

Morton again received wide attention as a consequence of Gould’s 1978 *Science* paper [6], particularly when a condensed version of the *Science* paper was included in Gould’s 1981 award-winning *The Mismeasure of Man* [15][[3]](#footnote-4). The specifics of Gould’s analyses are described in our main text, but their impact was immediate and long-lasting. Morton’s work became viewed primarily as a cautionary tale of how biased views found reification in supposedly objective data [2,3].

One of the only challenges to Gould’s interpretations of Morton came in a short paper by Michael [16] based on his undergraduate honor’s thesis [17]. Michael used Morton’s measurements to recalculate the population averages for the cranial capacities given in Morton [5] and also remeasured 201 crania in the Morton collection. Michael found that, “his [Morton’s] specific cranial measurements contain few errors,” [16]. However, Michael [16,17] did not report any individual comparisons of his measurements with Morton’s, leaving the critical question unanswered: regardless of the number of skulls mismeasured by Morton, what is their population affinity?

While we come to largely similar conclusions as Michael, his analysis does not support his findings[[4]](#footnote-5). Michael only deals with one of Morton’s many tables [5]. His remeasurements are compared to Morton’s shot data, which were explicitly assumed by Gould [6] to be accurate. Michael’s remeasurements are reported erroneously, lack specifics on individual comparisons, and are missing the key data on the population affinity of potentially mis-measured specimens. Michael’s recalculations [16] of the Morton means [5] are of questionable value given the incongruent samples but, overlooking that, reveal the racial pattern of errors expected by Gould [6], contra Michael [16]. Michael’s defense of Morton against Gould’s claims overlooks the most relevant charges made by Gould. Brace [2] and Cook [3] have faulted Gould for not citing Michael’s work [16] in his revised edition of *The Mismeasure of Man* [18]. Given the flaws in Michael, it is just as problematic to take Michael’s critique at face value as it is to take Gould’s analysis at face value.

Perhaps the best characterization of the response to Michael’s work [16] is that provided by Kitcher [19]: “virtually nobody has reacted to Michael’s article by seeing it as a refutation of Gould – with two major exceptions: it is used in this way in (Herrnstein and Murray 1994) [*The Bell Curve*] and is much ballyhooed by J. Philippe Rushton ....” This is an apt summary of the reaction, or lack thereof, to Michael’s work, which was not mentioned in the 1996 revised edition of Gould’s *Mismeasure* [cf. 2,3]. Kitcher also states that: “The truth of the matter is that Gould’s interpretations of Samuel Morton’s cranial data have been *questioned* by John S. Michael, who, as an undergraduate student at Macalester College, remeasured the skulls as part of an honors project (Michael 1988). It is not entirely evident that one should prefer the measurements of an undergraduate to those of a professional paleontologist whose own specialist work included some very meticulous measurements of fossil snails ... [particularly since there are only] relatively modest differences between Gould’s measurements and Michael’s.” [19, emphasis in original]. This might be a valid point, had Gould actually remeasured skulls from the Morton collection, but he did not – Gould relied on Morton’s measurements, which he reanalyzed. Instead, Kitcher assumed that Gould had remeasured the skulls, and this mistake illustrates the degree to which Gould's "plausible scenario" of Morton mismeasuring has been transformed into fact, and how little impact Michael's work has had [cf. 2-4].

Most recently, Brace [2] and Cook [3] made a number of salient points regarding Morton and Gould as part of their historical reviews of early physical anthropology in the New World, as does Buikstra [4] in her preface to a reprint of *Crania Americana*. Cook, “finds Gould’s arguments unpersuasive because it views Morton’s work through the lens of 20th-century quantitative sophistication,”[3], while Brace concludes that Gould’s critique of bias better fits Gould than Morton [2]. Both note the “presentism” of Gould’s historical analysis of Morton, as does Buikstra [4], but beyond summarizing Michael’s findings [16], neither offers specifics regarding Gould’s analysis [2,3]. Buikstra [4] provides a detailed biographical treatment of Morton, summarizes Michael’s findings [16], and faults Gould for mischaracterizing Morton. Brace [2] observes that some of the measurements developed by Morton are in use today, and Buikstra [4] notes that Morton’s approach to data is praiseworthy even by modern standards[[5]](#footnote-6). Cook observes that, “the Moundbuilder myth was a species of racism, and we should celebrate Morton for undermining it,” [3]. To the contrary, though, her “experience in teaching Gould’s paper to undergraduates has been that Gould unfairly brands Morton as a racist,”[3].

**References**

1. Stanton W (1960) The Leopard's Spots. Chicago: The University of Chicago Press.

2. Brace CL (2005) "Race" Is a Four-Letter Word: The Genesis of the Concept. New York: Oxford University Press.

3. Cook DC (2006) The old physical anthropology and the New World: A look at the accomplishments of an antiquated paradigm. In: Buikstra JE, Beck LA, editors. Bioarchaeology: The Contextual Analysis of Human Remains. Amsterdam: Elsevier. pp. 27-71.

4. Buikstra JE (2009) Introduction to the 2009 Reprint Edition of Crania Americana. Davenport, Iowa: Gustav’s Library. pp. i-xxxvi.

5. Morton SG (1849) Catalogue of Skulls of Man and the Inferior Animals, Third Edition. Philadelphia: Merrihew and Thomson Printers.

6. Gould SJ (1978) Morton's ranking of races by cranial capacity: Unconscious manipulation of data may be a scientific norm. Science 200: 503-509.

7. Morton SG (1839) Crania Americana; or, A Comparative View of the Skulls of Various Aboriginal Nations of North and South America: to Which is Prefixed an Essay on the Varieties of the Human Species. Philadelphia: J. Dobson.

8. Gruber JW (1981) American archaeology and physical anthropology in historical perspective. Am J Phys Anthropol 56: 473-482

9. Morton SG (1844) Crania Aegyptiaca; or, Observations on Egyptian Ethnography, Derived from Anatomy, History, and the Monuments. Philadelphia: John Penington.

10. Meigs JA (1857) Catalogue of Human Crania in the Collection of the Academy of Natural Sciences of Philadelphia: Based upon the Third Edition of Dr. Morton's "Catlogue of Skulls", &c. Philadelphia: The Academy of Natural Sciences of Philadelphia.

11. Renschler E,Monge JM (2008) The Samuel George Morton cranial collection: Historical significance and new research. Expedition 50: 30–38.

12. Nott JC, Glidden GR (1855) Types of Mankind. Philadelphia: Lippincott, Grambo and Co.

13. Erickson P (1974) Racial determinism and nineteenth century anthropology. Man 9: 489-491.

14. Brace CL (1982) The roots of the race concept in American physical anthropology. In: Spencer F, editor. A History of American Physical Anthropology, 1930 - 1980. New York: Academic Press. pp. 11-29.

15. Gould SJ (1981) The Mismeasure of Man. New York: W. W. Norton and Company.

16. Michael JS (1988) A new look at Morton's craniological research. Curr Anthropol 29: 349-354.

17. Michael JS (1986) An Analysis of Samuel G. Morton’s Catalog of the Skulls of Man and the Inferior Animals, Third Ed., Based on a Remeasurement of a Random Sample of the Morton Collection of Human Crania. Unpublished Undergraduate Honor’s Thesis, Department of Geology, Macalester College, St. Paul, Minnesota.

18. Gould SJ (1996) The Mismeasure of Man, Revised Edition. New York: W.W. Norton and Company.

19. Kitcher P (2004) Evolutionary theory and the social uses of biology. Biol Philos 19: 1-15.

1. Stanton [1] quotes Morton, in a much later letter, as saying he supported polygenesis, “and in Crania Americana my position is the same, though more cautiously worded.” [↑](#footnote-ref-2)
2. In *Crania Aegyptiaca*, Morton referred to slavery as “inhuman traffic” [9]. [↑](#footnote-ref-3)
3. Our analysis focuses on Gould’s 1978 *Science* paper [6], as that is the primary and most detailed publication of his work on Morton. The Morton section of Gould’s 1981 *The Mismeasure of Man* [15] is a summary of his 1978 paper, and no changes to this section were made for the 1996 revised version of *Mismeasure* [18]. [↑](#footnote-ref-4)
4. For specifics, see http://www.stanford.edu/~lewisjas/Morton.html. [↑](#footnote-ref-5)
5. Morton’s description of his measurements is detailed, he documented his measurement error, and he published his actual measurements, attributes often missing from modern publications. [↑](#footnote-ref-6)
