# Supplementary material for: The Mismeasure of Science: Stephen Jay Gould versus Samuel George Morton on Skulls and Bias
Source: PLoS Biol. 2011 Jun 7;9(6):e1001071. doi: 10.1371/journal.pbio.1001071 (PMC3110184; doi:10.1371/journal.pbio.1001071)
Supplement: Text S2 — Materials and methods. (PDF) [file pbio.1001071.s002.pdf]

TEXT S2: MATERIALS AND METHODS

Gould suggested that Morton, influenced by racial bias, might have mismeasured the capacity of skulls when using seeds [1]. While Morton's measurements of cranial capacity reported in 1839 were done by filling crania with seed (via a method he described in extreme detail [2]), he soon became dissatisfied with the repeatability of this method: "there is sufficient diversity to occasion considerable variation in the results of several successive measurements of the same head, especially when taken by different persons. This variation was sometimes not less than three or four cubic inches; making it desirable to use some other bodies in place of the pepper seeds" [3]. Morton then switched to using lead shot (circa 1/8" diameter) and found much greater repeatability: "in six successive measurements of the same skull, the results did not vary by more than half a cubic inch" [3]. While an assistant performed some of the seed-based measurements, Morton did all of the shot-based measuring of the crania himself [4].

Sample

Morton's 1839 *Crania Americana* [2] reported seed-based measurements, taken with the aid of an assistant, for 158 individual crania. All 158 are Native American, so it is not possible to test directly the possible racial bias of Morton's seed-based measurements. Even so, we remeasured 51 of these 158 Native American crania (32%). Our sample size is limited because many of the Native American crania are no longer in the Morton Collection. These remeasurements provide data on the general accuracy of the seed measurements reported by Morton.

While the seed-based method would seem to be more susceptible to bias [1], the shot-based method is unlikely to be immune from it: there is still a judgment of when the cranium is full, and the choice of how carefully to pack the shot into the cranium. Of the 670 crania measured by Morton with shot, we selected 308 (46%) to remeasure (Table S1) based on the completeness of the specimens and the lack of soft tissue. A power test indicates that only 208 specimens would need to be remeasured to detect differences at the 0.05 alpha level, so our sample size is sufficient.

Table S1. Comparison of our sample with that of Morton's original 1849 sample by population group. The "% of Morton" column reports what percentage our group sample is of Morton's. "Whites" refers to European and Caucasian populations, "Blacks" to African and African

Diaspora populations. These groups are not suggested to have any particular biological significance, but they roughly align with the biases Gould attributed to Morton.

	# of Crania Measured		
**Group**	**Morton (1849)**	**Our Study**	**% of Morton**
Whites	43	24	56%
Blacks	201	76	38%
N. America	174	90	52%
S. America	162	56	35%
Asia/Pacific	81	54	67%
Australia	9	8	89%
Total	*670*	*308*	*46%*

Our "remeasurement" sample includes crania from 73 geographic and ethnic populations, and contains some pathological specimens (e.g., artificial cranial deformation, dwarfism, syphilis, sword wounds) measured by Morton. While most studies exclude pathological specimens, our goal was to compare our measurements with those of Morton, so any skull he measured was appropriate for us to include. Morton classified crania according to a scheme of 22 "families" grouped into five or more "races" [2]. Our grouping scheme is based on Morton's classification, as a key test is whether Morton's errors were non-random with respect to his racial groupings. Our categories were Native Americans exclusive of Peruvians; Peruvians; whites (crania Morton considered Caucasian, including "Pelasgics" but excluding other Egyptians); blacks (African/African Diaspora exclusive of Egyptians); Egyptians; Australians; "Mongolians" (Morton's term/category); and "Malay" (again Morton's term/category). This was done as Morton's hypothesized bias would be positive for ancient (non-"Negro") Egyptians, while negative for other Africans, and positive for Peruvians, while negative for other Native Americans, due to the monuments associated with Incas and Egyptians [5,6]. As a simpler alternative, we also divided the sample by continent of origin (though including African diaspora specimens with Africans and Caucasian-Americans with Europeans).

Measurement Method

We measured the cranial capacity of the skulls by using molded acrylic balls [7]. Any large openings to the endocranium (e.g., jugular foramen, orbits) were first plugged with cotton. The cranium was placed in a plastic retaining tray with the foramen magnum facing up and the splanchocranium directed away from the measurer. Six-

millimeter diameter (0.1 cm^3) solid precision molded non-compressible acrylic balls (Greene Plastics Corporation, Hope Valley, Rhode Island) were poured into the foramen magnum until the balls would no longer flow freely into the cranium. The funnel was then removed from the foramen magnum, and the cranium gently shaken in both hands to level the balls inside. More balls were then poured into the cranium until the balls again stopped flowing, and the gentle shaking repeated. This process was continued until they overflowed into the funnel and out of the foramen magnum. To avoid deliberate or unconscious packing of the acrylic balls, the measurer would not place their fingers into the neurocranium to pack or push on the balls, but only shake intermittently. Once the cranium overflowed, the excess balls between the occipital condyles were leveled with a probe.

When the cranium was full, the balls were transferred into a graduated cylinder. The mass of the balls was measured using a triple-beam balance, and measurements were taken in increments of 0.025 grams. Mass was used rather than volume to avoid the error associated with visually reading a meniscus of spheroid objects as well as fluctuations due to differences in the packing of the balls in the cylinder. Instead, a ratio that relates volume and mass was experimentally determined by weighing known volumes of balls. This ratio was then used to calculate total cranial volume from the mass of the balls, as follows: [mass of balls (g)]/ 0.70517 = [cranial capacity (cm^3)]. This approach also lessens the awareness of the investigator of what specific volumes are being produced during the measuring operation.

After measuring a specimen, approximately half of the volume of balls in the graduated cylinder were poured into the previously used container, and the other half into a separate container. This was done to ensure the measurer was not biased towards trying to simply pack the previous volume of balls into the same cranium to be remeasured. Care was used throughout this process to protect the cranium from damage and to make sure all the acrylic balls were properly transferred from the cranium to the graduated cylinder. This entire process was repeated three times for each cranium, resulting in three different volume measurements, which allowed us to determine intraobserver measurement error. The three volumes were then averaged together to find a mean volume for each cranium, and it was this number that was used as the

experimentally determined cranial capacity. One observer (JEL) took the measurements for all crania but seven (six of which were a mix between JEL and MRM, and one of which was MRM only).

Our measurement error was calculated based on our three repeated measures of each cranium's capacity, in cm^3, and the percentage error [8,9] is on average 0.35% with a range of 0.04% to 1.48% (only two outlier specimens had errors > 1%). The sample size for the measurement error is 290 specimens, as the individual values (though not the means) were lost for 18 specimens.

Our measurements were taken in cm^3, but were converted to in^3 for comparison with Morton's measurements. The conversion was done in this direction because in^3 are larger units than cm^3, so converting Morton's in^3 to cm^3 would create more rounding error than the reverse. In any case, we also converted Morton's in^3 to cm^3 (data not shown) and doing so does not alter the results presented here. We rounded our in^3 values to the nearest whole number, as most of Morton's cranial capacity measurements are also reported in this fashion. Fortunately, the magnitude of the differences introduced by rounding and conversion issues is relatively small (less than 1%) and does not impact the results.

Comparing Our Method with Morton's

Before examining the question of whether Morton mismeasured crania, we must first establish the correspondence between cranial capacities measured using our acrylic ball method and those produced by Morton's methods. For Morton's seed-based measurements (the "seed" sample [2]), his mean for the 51 crania we remeasured is 1318 cm^3, whereas ours is 1301 cm^3, a difference of about 1% or 1 in^3. For Morton's shot-based measurements (the "shot" sample [4]), Morton's mean for the 308 crania we remeasured is 1350 cm^3, whereas ours is 1299 cm^3, a difference of about 4% or 3 in^3. When our individual measurements are subtracted from Morton's individual measurements, the median (and mean) difference is 51 cm^3 (see Dataset S2). In general, then, our measurement method yields cranial capacities that are circa 50 cm^3 less than, or are on average 96% of, those produced by Morton's. This difference is quite consistent across the sample, as evidenced by our ability to predict Morton's measurements from ours with an r^2 of 0.96 ($p < 0.0001$) using a linear regression model (Figure S1).

Figure S1. Linear regression of our cranial capacity measurements against Morton's 1849 shot-based cranial capacity measurements [4], both in in^3.

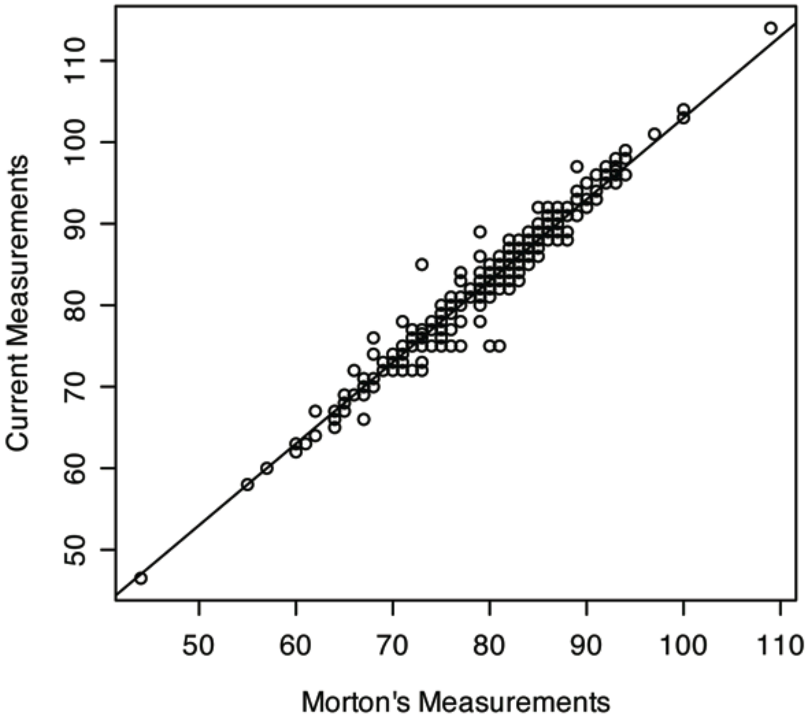

Statistical Methods

All statistical analyses were performed using the open source program 'R' (http://www.r-project.org), and the actual R code used is given below (Appendix I - R Code Used for Statistical Analyses). We used linear regression to examine differences between our measurements and Morton's due to our measurement methods (Figure S1). A normal quantile-quantile plot was then used to help identify outlier crania (those Morton may have mismeasured). Such an analysis plots the standardized residual of each specimen in the regression according to its magnitude (Figure S2). We also examined the simple percentage differences between our measurements and Morton's to identify possible errors on his part (Tables S2-3).

Figure S2. Standardized residuals for each specimen (their distance from the regression line) plotted by quantiles. The farther a point is from zero on the x-axis, the farther it is from the middle of the distribution. Positive residuals indicate that Morton overmeasured, negative

residuals indicate that he undermeasured. Morton's data are his shot-based measurements [4]. The farthest outliers are labelled by specimen number.

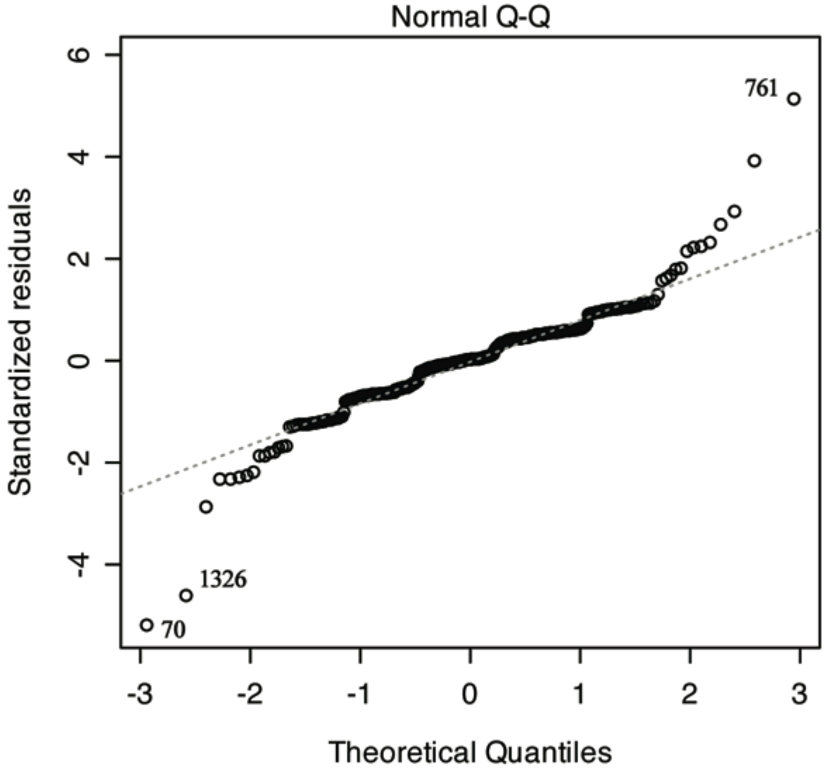

Table S2. Crania mismeasured by Morton (and/or his assistant) with seed, using our measurements as the "gold standard". All are Native American, as those are the only crania for which Morton reported individual seed measurements in 1839 [2]. Our capacity measurements ("Current") have been adjusted to account for the average difference (about 1%) produced by the difference in our method versus Morton's seed method. "Difference" is Morton's measurement relative to ours. "Measure Error" is our measurement error based on three repeated measurements of each cranium's capacity.

		Cranial Capacity (in^3)			
**Specimen #**	**Population**	**Current**	**Morton**	**Difference**	**Measure Error**
76	Peruvian	61	64	+5%	0.3%
54	Osage	79	83	+5%	0.4%
100	Peruvian	65	60	-8%	0.3%
96	Peruvian	77	68	-12%	0.4%

Table S3. Crania mismeasured by Morton with shot, using our measurements as the "gold standard". Our capacity measurements ("Current") have been adjusted to account for the average difference (about 4%) produced by the difference in our method versus Morton's shot method [4]. "Difference" is Morton's measurement relative to ours. Specimens with a percentage difference of greater than 5.5% (more than 2.5 standard deviations from the mean percentage difference) are clear outliers and thus are considered to have been mismeasured by Morton.

"Measure Error" is our measurement error based on three repeated measurements of each cranium's capacity.

		Cranial Capacity (in^3)			
**Specimen #**	**Population**	**Current**	**Morton**	**Difference**	**Measure Error**
761	Egyptian Copt	76	85	+12%	0.5%
754	Seminole	82	89	+9%	0.2%
994	Native African	71	76	+7%	0.4%
1435	Peruvian	70	66	-6%	0.3%
949	Arickaree	80	75	-6%	0.2%
1326	Peruvian	83	75	-10%	0.5%
70	Chetimaches	84	75	-11%	0.5%

To test whether Morton's errors in cranial capacity measurements were randomly distributed by population, we used a binomial probability analysis of quantile groupings of the percentage differences between our measurements and Morton's. Using our measurements as a baseline, our preferred quantile scheme was to divide the sample into three groups of crania: I, Morton overestimated (>3% greater than our measurement); II, Morton measured about right (≤ 3% different from our measurement); and III, Morton underestimated (>3% less than our measurement). The threshold of 3% was chosen both because it appeared to be a "break point" in the rank order distribution of absolute percent differences between our measurements and Morton's (see Dataset S2), and also because differences of greater than 3% correspond to more than 2 in^3. Given the difference in methods between our measurements and Morton's, unit conversions, rounding, and a measurement error of about 0.3%, it is difficult to attribute a difference of 2 in^3 (or less) to error on Morton's part. These quantiles were then sorted by population to form a "quantile by population" table (that specifies, for example, the number of African specimens in quantile III as one cell). Our preferred population scheme is based on Morton's categories and is as follows: Native Americans exclusive of Peruvians; Peruvians; whites (crania Morton considered Caucasian, including "Pelasgics" but excluding other Egyptians); blacks (African/African Diaspora exclusive of Egyptians); Egyptians; Australians; "Mongolians" (Morton's term/category); and "Malay" (again Morton's term/category). The binomial function was applied to this quantile/population table to identify cells with values that were either significantly larger or smaller than would be expected by chance. This identifies whether, for example, more white crania

were overestimated by Morton than would be expected if his errors were randomly distributed by population.

The results of the quantile – binomial analysis depend in part on the choice of population and quantile groupings. We therefore explored the impact of different choices in each regard. Our alternate population grouping was to consider the sample by continent of origin, though including African diaspora specimens with Africans and pooling all Caucasians with Europeans. Our alternate quantile scheme was to use five groups of crania: I, specimens Morton overestimated by ≥5%; II, specimens slightly overestimated by Morton (1-4%); III, specimens measured accurately by Morton (±1%); IV, specimens slightly underestimated by Morton (1-4%); and V, specimens Morton underestimated (≥5%). Both quantile schemes were applied to the entire sample using both population groupings.The five quantile scheme applied to Morton's population groupings finds that Morton underestimated fewer "black" crania than expected by chance (data not shown). Using our simpler "continental" population grouping scheme, with both three and five quantile divisions, also shows that Morton underestimated fewer "black" crania than expected by chance (data not shown). Furthermore, we also used a two quantile scheme applied to just the mismeasured crania, rather than the entire sample of percentage differences, but no cells approached significance.

References

1. Gould SJ (1981) The Mismeasure of Man. New York: W. W. Norton and Company.
2. Morton SG (1839) Crania Americana; or, A Comparative View of the Skulls of Various Aboriginal Nations of North and South America: to Which is Prefixed an Essay on the Varieties of the Human Species. Philadelphia: J. Dobson.
3. Morton SG (1841) Minutes of Stated Meeting, April 6, 1841. Proceedings of the Academy of Natural Sciences of Philadelphia 1: 7-8.
4. Morton SG (1849) Catalogue of Skulls of Man and the Inferior Animals, Third Edition. Philadelphia: Merrihew and Thomson Printers.
5. Stanton W (1960) The Leopard's Spots. Chicago: The University of Chicago Press.
6. Gould SJ (1978) Morton's ranking of races by cranial capacity: Unconscious manipulation of data may be a scientific norm. Science 200: 503-509.
7. Michael JS (1988) A new look at Morton's craniological research. Current Anthropology 29: 349-354.
8. Utermohle CJ, Zegura SL (1982) Intra- and interobserver error in craniometry: A cautionary tale. American Journal of Physical Anthropology 57: 303-310.

9. Utermohle CJ, Zegura SL, Heathcote GM (1983) Multiple observers, humidity, and choice of precision statistics: Factors influencing craniometric data quality. American Journal of Physical Anthropology 61: 85-95.

Appendix I - R Code Used for Statistical Analyses

Note that the following "R" code (http://www.r-project.org) also includes additional analyses beyond those reported here.

To load data file:

```
> morton = read.table("path to location of data file on your computer/Morton Data File
name.extension", header=T, sep = ",")
```

Linear regression analyses (based on raw values, not corrected values):

```
> lm(formula = morton$CCAP ~ morton$Morton.IC)

Call:
lm(formula = morton$CCAP ~ morton$Morton.IC)

Coefficients:
    (Intercept)  morton$Morton.IC
        1.8667           0.9397
> summary(lm(formula = morton$CCAP ~ morton$Morton.IC))

Call:
lm(formula = morton$CCAP ~ morton$Morton.IC)

Residuals:
   Min      1Q  Median      3Q     Max
-8.7433 -0.9659 -0.1173  0.9437  8.6539

Coefficients:
                 Estimate Std. Error t value Pr(>|t|)
(Intercept)        1.8667     0.9114   2.048   0.0414 *
morton$Morton.IC   0.9397     0.0110  85.423   <2e-16 ***
---
Signif. codes:  0 '***' 0.001 '**' 0.01 '*' 0.05 '.' 0.1 ' ' 1

Residual standard error: 1.692 on 306 degrees of freedom
Multiple R-squared: 0.9598,       Adjusted R-squared: 0.9596
F-statistic:  7297 on 1 and 306 DF,  p-value: < 2.2e-16
```

Binomial probability analyses:

-based on continent, with five % difference divisions:

```
> MCTable=table(morton$Quantile.I, morton$Pop)
> MCTable

      1  2  3  4  5  6
 I    1  5  2  1  0  0
 II  11 34 31 17 18  1
```

```
  III  5 20 23 11 16  3
  IV   7 15 31 24 20  4
  V    0  2  3  3  0  0
> Ecnt <- outer(rowSums(MCTable), colSums(MCTable), "*") / sum(MCTable)
> Eprop <- Ecnt/sum(MCTable)
> Oprop <- MCTable / sum(MCTable)
> Cm <- ceiling(Oprop-Eprop)
> BP <- pbinom(MCTable-Cm, sum(MCTable), Eprop)
> BP <- Cm-BP
> BP

              1           2           3           4           5           6
  I    0.50445560  0.07405398 -0.51028377 -0.51272499 -0.20556811 -0.79147658
  II   0.26008927  0.12279507 -0.41932235 -0.26189151 -0.40790441 -0.21170941
  III -0.43154172  0.46257048  0.51299148 -0.23891955  0.29599099  0.33037586
  IV  -0.46963305 -0.01908103  0.41443944  0.11096284  0.32000147  0.26873945
  V   -0.53579250  0.58777669  0.41426211  0.17951273 -0.24517065 -0.81231886
```

-based on continent, with three % difference divisions:

```
> MCTable=table(morton$Quantile.II, morton$Pop)
> MCTable

      1  2  3  4  5  6
  I   12 39 33 18 18  1
  II   5 20 23 11 16  3
  III  7 17 34 27 20  4
> Ecnt <- outer(rowSums(MCTable), colSums(MCTable), "*") / sum(MCTable)
> Eprop <- Ecnt/sum(MCTable)
> Oprop <- MCTable / sum(MCTable)
> Cm <- ceiling(Oprop-Eprop)
> BP <- pbinom(MCTable-Cm, sum(MCTable), Eprop)
> BP <- Cm-BP
> BP

              1           2           3           4           5           6
  I    0.23783398  0.05207250 -0.37781083 -0.22307531 -0.27757188 -0.17731527
  II  -0.43154172  0.46257048  0.51299148 -0.23891955  0.29599099  0.33037586
  III -0.38372644 -0.02358664  0.37035876  0.06523157  0.44974689  0.31482855
```

-based on revised 'racial' groupings, with three % difference divisions:

```
> MCTable=table(morton$Quantile.II, morton$DD.Pop)
> MCTable

      1  2  3  4  5  6  7  8
  I   21 30  6 35 16  2 10  1
  II  15 16  2 23 11  4  4  3
  III 19 11  5 34 27  1  8  4
> Ecnt <- outer(rowSums(MCTable), colSums(MCTable), "*") / sum(MCTable)
> Eprop <- Ecnt/sum(MCTable)
> Oprop <- MCTable / sum(MCTable)
> Cm <- ceiling(Oprop-Eprop)
> BP <- pbinom(MCTable-Cm, sum(MCTable), Eprop)
> BP <- Cm-BP
> BP
```

```
            1          2          3          4          5          6          7          8
 I   -0.50329567  0.06399594  0.40298596 -0.46360482 -0.14301133 -0.48064361  0.36497058 -
0.17731527
 II   0.42179690  0.37283878 -0.35959488 -0.52945920 -0.28288505  0.10380686 -0.34430439
0.33037586
 III -0.51701506 -0.01676672  0.48773502  0.42149869  0.04579653 -0.29075312  0.51818910
0.31482855
```

-based on revised 'racial' groupings, with five % difference divisions:

```
> MCTable=table(morton$Quantile.I, morton$DD.Pop)
> MCTable

     1  2  3  4  5  6  7  8
 I    2  2  2  2  1  0  0  0
 II  19 28  4 33 15  2 10  1
 III 15 16  2 23 11  4  4  3
 IV  19  9  5 31 24  1  8  4
 V    0  2  0  3  3  0  0  0
> Ecnt <- outer(rowSums(MCTable), colSums(MCTable), "*") / sum(MCTable)
> Eprop <- Ecnt/sum(MCTable)
> Oprop <- MCTable / sum(MCTable)
> Cm <- ceiling(Oprop-Eprop)
> BP <- pbinom(MCTable-Cm, sum(MCTable), Eprop)
> BP <- Cm-BP
> BP

            1           2           3           4           5           6           7           8
 I    0.477884982  0.496559269  0.056137989 -0.495750697 -0.531608908 -0.814962316 -
0.525434908 -0.791476579
 II  -0.467259576  0.066442125 -0.488539638 -0.512882674 -0.167584108 -0.531632461
0.281725117 -0.211709412
 III  0.421796896  0.372838781 -0.359594878 -0.529459203 -0.282885047  0.103806864 -
0.344304391  0.330375859
 IV   0.441171779 -0.008861967  0.422752325  0.464493886  0.082554336 -0.330783785
0.433553618  0.268739449
 V   -0.238855928  0.436023402 -0.713304013  0.427971122  0.166799537 -0.833708158 -
0.564418484 -0.812318859
```

-seventeen outliers, based on revised 'racial' groupings, with five % difference divisions:

-here we created a new file with only the 17 outliers, but this can easily be done from with R as well

```
> seventeen = read.table("path to location of data file on your computer/Seventeen Outliers File
name.extension", header=T, sep = ",")
> MCTable=table(seventeen$Quantile.I, seventeen$DD.Pop)
> MCTable

   1 2 3 4 5
 I 2 2 2 2 1
 V 0 2 0 3 3
> Ecnt <- outer(rowSums(MCTable), colSums(MCTable), "*") / sum(MCTable)
> Eprop <- Ecnt/sum(MCTable)
> Oprop <- MCTable / sum(MCTable)
> Cm <- ceiling(Oprop-Eprop)
```

```
> BP <- pbinom(MCTable-Cm, sum(MCTable), Eprop)
> BP <- Cm-BP
> BP

          1          2          3          4          5
 I  0.2864554 -0.6430617  0.2864554 -0.4930811 -0.3561895
 V -0.3797534  0.5760651 -0.3797534  0.4246968  0.2892696
```

-the same table results for 3 % difference divisions.

Power Analysis:

```
> pwr.r.test(n=308, r=0.9805031, sig.level=0.05, alternative="two.sided")

     approximate correlation power calculation (arctangh transformation)

              n = 308
              r = 0.9805031
      sig.level = 0.05
          power = 1
    alternative = two.sided

> d= (mean(mortoncorr$CCAP)-mean(mortoncorr$MORTONCCA,
na.rm=T))/sd(mortoncorr$MORTONCCA, na.rm=T)
> d
[1] -0.3518261
> pwr.t.test(d=d, power= .999, sig.level=0.05, type="one.sample", alternative="two.sided")

     One-sample t test power calculation

              n = 207.9799
              d = 0.3518261
      sig.level = 0.05
          power = 0.999
    alternative = two.sided
```

Stripchart code:

```
> plot(morton$JL.CCAP.CORRECTED, rep(1,length(morton$JL.CCAP.CORRECTED)), ylab="",
xlab="", pch=0, ann=FALSE, axes=FALSE, ylim=c(0,4))
> box()
> ccapaxis=round(morton$CCAP)
> ccapaxis=round(morton$JL.CCAP.CORRECTED)
> axis(ccapaxis,side=1)
> points(morton$Morton.IC, rep(3, length(morton$JL.CCAP.CORRECTED)))
> segments(morton$JL.CCAP.CORRECTED,1,morton$Morton.IC,3)
> my.names <- c("Remeasure","Morton")
> axis(2, at=c(1,3), labels=my.names)
```
